# EBV-associated leiomyosarcoma in an immunocompromised child: A unique intracranial case with genomic study

**DOI:** 10.1016/j.ijscr.2025.111073

**Published:** 2025-02-18

**Authors:** Wiem Ben Makhlouf, Rim Kallel, Roeya Kolsi, Sana Kmiha, Khalil Ayadi, Tahya Boudawara

**Affiliations:** a Anatomy and Pathological Cytology Laboratory, Habib Bourguiba University Hospital, Sfax, Tunisia; b Pediatrics Department, Hedi Chaker University Hospital, Sfax, Tunisia; c Neurosurgery Department, Habib Bourguiba University Hospital, Sfax, Tunisia

**Keywords:** EBV-associated tumors, Leiomyosarcoma, Immunodeficiency, Childhood tumors

## Abstract

**Introduction and importance:**

Epstein-Barr virus (EBV) is a common virus infecting more than 90 % of the adult population, typically without symptoms. While most infections remain asymptomatic, EBV is associated with over 200,000 new cancer cases annually. It is linked to several malignancies, including leiomyosarcoma (LS) in immunocompromised patients, a rare occurrence with fewer than 100 new cases per year globally. This report highlights the case of an EBV-associated intracranial leiomyosarcoma in a 4-year-old immunodeficient child.

**Case presentation:**

A 4-year-old girl with a history of primary immune deficiency and multiple infections presented with febrile dyspnea. Imaging revealed a right temporo-parietal brain mass, which increased in size over 50 days. Surgical excision was performed, and histological examination showed a tumor with smooth muscle cell characteristics. Immunohistochemical analysis was positive for vimentin and CD99, while EBV genome presence was confirmed by in situ hybridization. The final diagnosis was EBV-associated malignant smooth muscle tumor. The postoperative course was favorable, and chemotherapy was not indicated.

**Clinical discussion:**

Leiomyosarcoma is extremely rare in immunocompetent children but more common in immunocompromised individuals, where EBV infection plays a significant role in tumor development. Although EBV-related leiomyosarcomas occur more frequently in immunodeficient children, intracranial cases are exceptionally rare. These tumors are often challenging to diagnose due to their undifferentiated appearance. The detection of EBV DNA using in situ hybridization is crucial for confirming the diagnosis. While EBV-associated leiomyosarcomas generally respond well to therapy, the optimal treatment remains unclear, with surgery and radiotherapy being the primary approaches.

**Conclusion:**

EBV-associated smooth muscle tumors are rare but increasing in incidence among immunocompromised patients. Early recognition of EBV infection in smooth muscle tumors, especially in children with immune deficiencies, is vital for diagnosis. Histological and molecular examination, including in situ hybridization, is essential to confirm the presence of EBV. Treatment typically involves complete surgical excision, with chemotherapy's role still uncertain.

## Highlights

• EBV-associated SMTs make up 3-17% of cancers in immunocompromised individuals, but less than 100 new EBV-LS cases occur annually.
• Diagnosing EBV-SMTs is challenging due to their undifferentiated appearance and difficulty distinguishing benign from malignant forms.
• In situ hybridization with EBER identifies the EBV genome in tumor cells.
• Immunosuppression, young age, and smooth muscle appearance should prompt pathologists to perform in situ hybridization for EBV detection.

## 1 Introduction

The Epstein-Barr virus (EBV) is a member of the herpes virus family that infects the majority of the population. It is estimated that over 90 % of the adult population is infected and is usually asymptomatic. First contact with the virus typically occurs in the first few months after birth and it persists in the infected organism until the host dies [1]. Yet, this seemingly innocent virus is responsible for over 200,000 new cases of cancer arising worldwide each year. Actually, EBV is aetiologically linked to more than nine distinct human tumors which are in decreasing incidence: B lymphoproliferative disease, Hodgkin lymphoma, Burkitt lymphoma, Diffuse large B cell lymphoma, Nasopharyngeal carcinoma (Epithelial cell), Gastric carcinoma, T and NK cells T/NK lymphoproliferative diseases, T/NK lymphomas/leukaemias, and Leiomyosarcoma. The latter is extremely rare, affecting only immunocompromised subjects, with incidence limited to less than 100 new cases per year worldwide [2]. Herein, we report a case of an intracranial EBV-associated leiomyosarcoma in a 4-year-old immunodeficient girl.

## 2 Presentation of case

A 4-year-old girl from a first-degree consanguineous marriage has been followed since the age of 17 months for a primary immune deficiency with a defect in the expression of HLA class 2 molecules, under antibiotic prophylaxis and regular infusion of venoglobulins every 3 weeks. The patient was hospitalized on several occasions for 4 episodes of gastroenteritis, 2 episodes of acute bronchiolitis, one episode of acute otitis media with *E. coli* and Pseudomonas, one episode of pulmonary Pneumocystis, and two episodes of bronchopulmonary aspergillosis.

The patient is currently presenting with febrile dyspnea and has been diagnosed with disseminated BCGitis, having received four courses of anti-tuberculosis therapy. A brain CT scan performed as part of the extension workup revealed a right temporo-parietal collection with intense parietal enhancement, surrounded by parenchymal edema, measuring 50 × *40 mm.* A puncture of this cystic mass was performed. The bacteriological examination and Koch's bacillus research were negative.

A follow-up CT scan was performed after 50 days and showed an increase in the size of the parieto-temporo-occipital collection, measuring 50 × 58 mm, with deviation of the midline, collapse of the lateral ventricle, and the appearance of exclusion hydrocephalus of the occipital horn. Surgical excision was performed.

Macroscopic examination revealed a cystic lesion measuring 4 × 3 cm, with a thick wall and blackish fluid content.

**Fig. 1:**
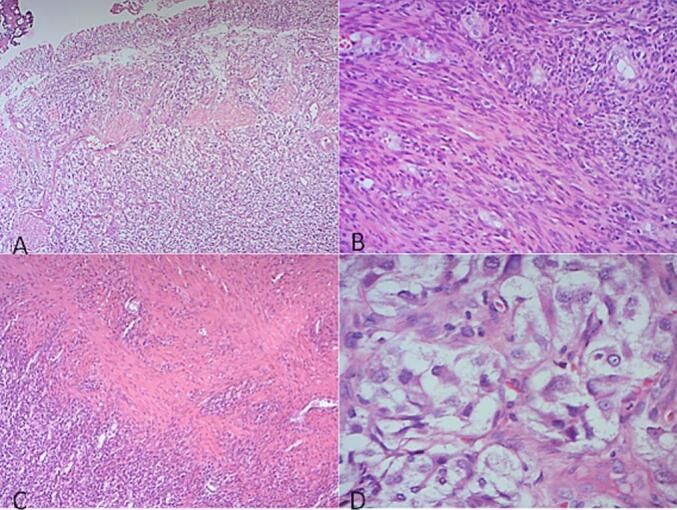
Histological features of the tumor. A: Intracystic proliferation of medium-sized round or spindle-shaped cells, demonstrating a hemangio-pericardial vascular system with capillary-like vessels. This vascular pattern is characteristic of the tumor's hypervascular nature (HE ×25). B/C: Proliferation of smooth muscle-like spindle cells arranged in tangled or storiform bundles. These cells exhibit features typical of leiomyosarcoma, with elongated nuclei and eosinophilic cytoplasm, reminiscent of smooth muscle cells (HE ×100). D: Nuclei of the tumor cells show moderate atypia, with some cells having a prominent nucleolus, indicative of cellular dysregulation. These histological features are typical of a malignant smooth muscle tumor (HE ×400).

On histological examination, the cystic wall is formed by a tumoral proliferation of medium-sized round or spindle-shaped cells arranged in small tangled bundles, sometimes with a storiform or, rarely, microcystic appearance. Tumor cells have poorly defined eosinophilic cytoplasm. Nuclei are elongated or oval, sometimes discretely nucleated, with moderate atypia. The mitotic index is estimated at 3 mitoses/10 CFG (Fig. 1).

The tumor proliferation is of variable density, alternating between hypercellular and pauci-cellular, edematous zones, with cells reminiscent of smooth muscle cells. The tumor is richly vascularized by capillary-type vessels of variable size, sometimes dilated and often branching, creating a hemangio-pericytoid-like appearance. There are a few foci of necrosis. The tumor is punctuated by a few lymphocytes and bordered by fibrino-cruoric material on the inner surface of the cyst (Fig. 1).

**Fig. 2:**
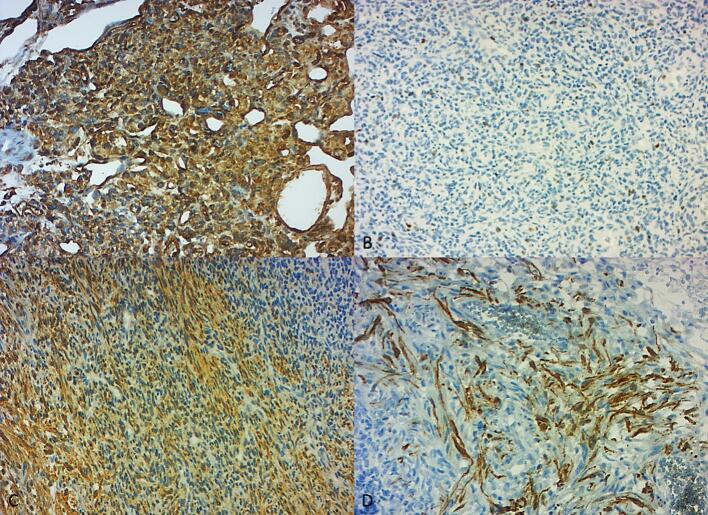
Immunohistochemical features of the tumor. A: Vimentin ×200: The tumor cells display diffuse and intense staining for vimentin, a marker for mesenchymal origin, confirming the smooth muscle differentiation of the cells. B: Ki67 × 200: Immunostaining for Ki67 shows a proliferation index estimated at 40 %, indicating a high rate of cell division, which correlates with the aggressive behavior of the tumor. C: AML × 100: Smooth muscle-like cells are highlighted with AML (Acute Myeloid Leukemia) antibody staining. This marker shows the presence of smooth muscle actin, supporting the diagnosis of a smooth muscle tumor. D: Desmin ×200: Staining for Desmin shows positive results in the smooth muscle-like tumor cells, further confirming the diagnosis of a malignant smooth muscle tumor and indicating smooth muscle differentiation.

An immunohistochemical study was performed with a wide panel of antibodies: Stat 6, Alpha-inhibin, EMA, LCA, INI 1, Vimentin, Ps100, CD34, GFAP, Chromogranin, Synaptophysin, Desmin, Bcl2, CD99, Keratin, CD10, Melan A, HHV8, HMB45, CD68, WT1, AML, Myogenin, MDM2, and Ki67. Tumor cells diffusely and intensely expressed vimentin and were weakly positive for CD99. AML and Desmin marked the aforementioned smooth muscle cells. Ki67 was estimated at 40 %. No loss of INI 1 was noted. Immunostaining was negative for other antibodies (Fig. 2).

In situ hybridization with the EBER probe showed nuclear expression. The final diagnosis was that of an EBV-associated malignant smooth muscle tumor proliferation occurring in an immunocompromised subject.

The patient's outcome following surgical excision was favorable, with no recurrence of the tumor observed during the 6-month follow-up period. Postoperative neurological assessments showed marked improvement, and the patient regained full cognitive and motor function. Given the patient's immune status, the multidisciplinary team opted not to pursue chemotherapy, a decision informed by the favorable prognosis observed in similar cases of EBV-LS. Continuous monitoring, including regular imaging and immune function assessments, was recommended to detect any potential recurrence or complications.

## 3 Discussion

Leiomyosarcoma (LS) is an extremely rare tumor in immunocompetent children, with fewer than 2 cases per 10 million children per year [3], and it shows no association with EBV. However, in immunodeficient children, smooth muscle tumors (SMT) are the second most common malignancy after non-Hodgkin lymphoma, and they are proven to be EBV-induced. EBV-associated SMT exhibit increasing numbers in the past decade, constituting 3 % to 17 % of all cancers in immunodeficient people [4,5]. Yet, the incidence of EBV-LS remains rare, limited to fewer than 100 new cases per year worldwide [2].

The increased prevalence of SMT in immunocompromised patients has been linked to EBV infection; all the reported cases of LS in immunodeficient patients that were tested by in situ hybridization were proven to be EBV genome-positive [2,4].

Most EBV-LS occur in children with AIDS and less commonly with congenital immunodeficiency or post-transplantation [4]. Its occurrence in adults is less frequent [6]. In all cases, this group of tumors seems to be conditioned by a deficient immune status.

Two new cases published in January 2023 and March 2023 made the exception; Tabor et al. [7] and Al-Trawnah et al. [8] reported a case of an intracranial EBV-LS in an immunocompetent 40-year-old male patient and a pancreatic EBV-LS in an immunocompetent 60-year-old female patient, respectively.

It is classic for tumors to occur in immunosuppressed patients at different rates and in aberrant sites compared to the general population. In immunocompetent patients, LS usually arises from the mesenchymal soft tissue of the retroperitoneum, uterus, and gastrointestinal tract [9]. In immunocompromised patients, the most common locations that have been found are the gastrointestinal tract, pulmonary system, and genitourinary system. More atypical sites are seen, such as the spleen, liver, skin, and foot in children, and the adrenal gland, liver, lymph nodes, pleura, pericardium, and skin in adults [3]. Intracranial location remains exceptional. It has been speculated that the cell of origin in primary intracranial leiomyosarcoma originates from smooth muscles in blood vessel walls [10] and that the parasellar location can be explained by the rich vascular network supplying the pituitary gland [11].

Recent studies, such as those by Badour et al. [12], have highlighted the neurological complications associated with viral infections like EBV, particularly in immunocompromised patients. EBV can cause encephalitis, leading to cognitive and motor deficits, and has been linked to autoimmune disorders like Guillain-Barré syndrome and multiple sclerosis. Additionally, EBV is associated with primary CNS lymphomas. Understanding these neurological effects can provide valuable insights into the pathophysiology of EBV-related tumors, such as leiomyosarcomas, in immunocompromised children.

Diagnosing an EBV-LS is difficult for the pathologist because of its undifferentiated histological appearance. The presence of smooth muscle-like eosinophilic spindle cells expressing smooth muscle actin on immunohistochemical study should point to the diagnosis of SMT; however, the association with EBV cannot be demonstrated by immunohistochemical study, as the immunohistochemical staining for EBV latent membrane antigen–1 is known to be negative in EBV-SMTs [13]. In situ hybridization with the EBER enables identifying the EBV genome in the tumoral cell [3].

At the molecular level, Epstein-Barr virus (EBV) has been shown to contribute to the development of tumors in immunocompromised hosts by inducing immune evasion and promoting cellular proliferation. EBV expresses latent genes, such as LMP-1 and EBNA, which can trigger oncogenic pathways, including the activation of NF-κB and the inhibition of apoptosis. This dysregulation of cell cycle control and apoptosis is thought to play a significant role in tumorigenesis, particularly in patients with an impaired immune response [14].

Besides, the discrimination between benign and malignant SMTs may prove very difficult. Even a rare number of mitoses and any degree of atypia may indicate aggressive behavior [15].

Although the vast majority of EBV-SMT cases were diagnosed as leiomyomas, most cases affecting the central nervous system have shown malignant histological features and have been classified as LMS based on their cytomorphological features and very high mitotic count [13].

Although the number of cases of LMS is increasing, there are still too few patients to formulate effective treatment regimens. Complete surgical removal, together with radiotherapy, is the mainstay treatment. The efficacy of chemotherapy is unclear. In practical terms, the choice of treatment depends on the patient's age, immune status, and, above all, the site of the tumor [3,4,16].

Unlike conventional LS, EBV-LS seems to respond better to therapy, and they have been hypothesized to exhibit a more favorable clinical prognosis and a better postoperative course [9,17].

## 4 Conclusion

EBV-SMT is a rare group of tumors that exhibit increasing rates in the last decade. The diagnosis of these tumors is difficult, but the presence of a cluster of factors such as immunodeficiency, especially in patients with AIDS, young age, and the smooth muscle appearance of the tumor cells, which express smooth muscle actin antibodies, should lead the pathologist to perform in situ hybridization to identify the EBV genome and confirm the diagnosis.

## 5 Methods

The work has been reported in line with the SCARE criteria.

## Consent

Written informed consent was obtained from the patient's parents/legal guardian for publication and any accompanying images. A copy of the written consent is available for review by the Editor-in-Chief of this journal on request.

## Ethical approval

The study is exempt from ethical approval in my institution.

## Guarantor

Wiem Ben Makhlouf

## Funding

We do not have a funding source.

## Author contribution

Wiem Ben Makhlouf and Rim Kallel: Conceptualization; Data curation; Investigation; Methodology; Writing - original draft

Roeya Kolsi and Sana Kmiha: Data curation, Formal analysis; Investigation

Khalil Ayadi and Tahya Boudawara: Project administration; Supervision; Validation

## Conflict of interest statement

There is no conflict of interest.
